# Aqua­(2,2′-bipyrid­yl)(pyrazine-2,6-dicarboxyl­ato)nickel(II) 1.25-hydrate

**DOI:** 10.1107/S1600536808034806

**Published:** 2008-10-31

**Authors:** Feng-Qin Wang, Yuan-Fang Zhang

**Affiliations:** a School of Materials and Chemical Engineering and Key Laboratory of Hollow Fiber, Membrane Materials & Membrane Processes, Tianjin Polytechnic University, Tianjin 300160, People’s Republic of China; b Middle School of Gaohu in Yinan, Shandong 276314, People’s Republic of China

## Abstract

The asymmetric unit of the title compound, [Ni(C_6_H_2_N_2_O_4_)(C_10_H_8_N_2_)(H_2_O)]·1.25H_2_O, contains two independent chemically identical Ni^II^ complex cations and two and a half solvent water mol­ecules. The Ni^II^ ions are in slightly distorted coordination environments. In the crystal structure, inter­molecular O—H⋯O and weak C—H⋯O hydrogen bonds link cations and water mol­ecules into a three-dimensional network. One of the three uncoordinated water molecules is half-occupied.

## Related literature

For related structures, see: Wang *et al.* (2006 ▶); Wang, Weng, *et al.* (2007 ▶); Wang, Zheng & Jin (2007 ▶); Wang, Zheng, *et al.* (2007 ▶ ▶, 2008 ▶); Wang, Mu *et al.* (2008 ▶).
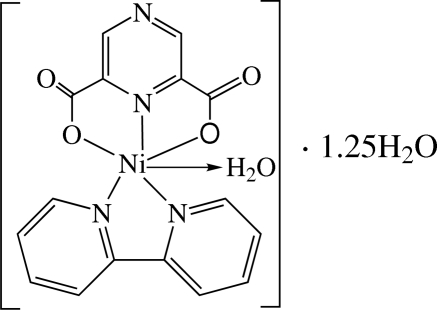


## Experimental

### 

#### Crystal data

[Ni(C_6_H_2_N_2_O_4_)(C_10_H_8_N_2_)(H_2_O)]·1.25H_2_O
*M* *_r_* = 421.5
Monoclinic, 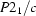

*a* = 10.7616 (12) Å
*b* = 14.8677 (17) Å
*c* = 21.933 (2) Å
β = 101.015 (2)°
*V* = 3444.6 (7) Å^3^
*Z* = 8
Mo *K*α radiation
μ = 1.17 mm^−1^
*T* = 294 (2) K
0.22 × 0.10 × 0.08 mm

#### Data collection

Bruker SMART CCD diffractometer
Absorption correction: multi-scan (*SADABS*; Sheldrick, 1996 ▶) *T*_min_ = 0.796, *T*_max_ = 0.871
19122 measured reflections
7030 independent reflections
4740 reflections with *I* > 2σ(*I*)
*R*_int_ = 0.035

#### Refinement

*R*[*F* ^2^ > 2σ(*F* ^2^)] = 0.037
*wR*(*F* ^2^) = 0.102
*S* = 1.04
7030 reflections
496 parameters
H-atom parameters constrained
Δρ_max_ = 0.31 e Å^−3^
Δρ_min_ = −0.26 e Å^−3^

### 

Data collection: *SMART* (Bruker, 1997 ▶); cell refinement: *SAINT* (Bruker, 1997 ▶); data reduction: *SAINT*; program(s) used to solve structure: *SHELXTL* (Sheldrick, 2008 ▶); program(s) used to refine structure: *SHELXTL*; molecular graphics: *SHELXTL*; software used to prepare material for publication: *SHELXTL*.

## Supplementary Material

Crystal structure: contains datablocks I, global. DOI: 10.1107/S1600536808034806/lh2711sup1.cif

Structure factors: contains datablocks I. DOI: 10.1107/S1600536808034806/lh2711Isup2.hkl

Additional supplementary materials:  crystallographic information; 3D view; checkCIF report

## Figures and Tables

**Table 1:** Selected bond lengths (Å)

Ni1—N1	1.986 (2)
Ni1—N3	2.047 (3)
Ni1—O5	2.059 (2)
Ni1—N4	2.067 (3)
Ni1—O3	2.143 (2)
Ni1—O1	2.184 (2)
Ni2—N5	1.986 (2)
Ni2—N8	2.044 (2)
Ni2—O10	2.068 (2)
Ni2—N7	2.072 (2)
Ni2—O6	2.125 (2)
Ni2—O8	2.175 (2)

**Table 2:** Hydrogen-bond geometry (Å, °)

*D*—H⋯*A*	*D*—H	H⋯*A*	*D*⋯*A*	*D*—H⋯*A*
C32—H32⋯O10	0.93	2.48	3.033 (4)	118
C31—H31⋯O13^i^	0.93	2.53	3.384 (11)	153
C29—H29⋯O6^ii^	0.93	2.53	3.318 (4)	143
C26—H26⋯O6^ii^	0.93	2.54	3.329 (4)	143
C14—H14⋯O11^i^	0.93	2.43	3.275 (5)	151
C9—H9⋯O1^iii^	0.93	2.47	3.281 (5)	146
C7—H7⋯O5	0.93	2.50	3.000 (4)	114
O13—H13*A*⋯O13^iv^	0.86	1.73	2.26 (2)	118
O12—H12*B*⋯O13	0.85	1.79	2.636 (12)	179
O12—H12*A*⋯O3	0.85	2.33	2.856 (4)	121
O11—H11*B*⋯O8	0.85	2.06	2.912 (3)	177
O11—H11*A*⋯O7^v^	0.85	2.07	2.921 (4)	178
O10—H10*B*⋯O4^vi^	0.86	1.85	2.699 (3)	170
O10—H10*A*⋯O2^vii^	0.85	1.84	2.694 (3)	178
O5—H5*B*⋯O7^viii^	0.85	1.85	2.686 (3)	169
O5—H5*A*⋯O9^ix^	0.85	1.78	2.621 (3)	173

Symmetry codes: (i) 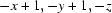; (ii) 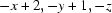; (iii) 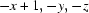; (iv) 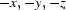; (v) 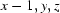; (vi) 
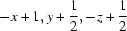
; (vii) 
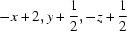
; (viii) 
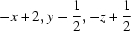
; (ix) 
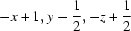
.

**Fig. 1.**
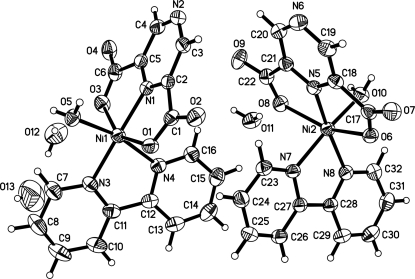
The asymmetric unit of the title complex. Dispacement ellipsoids are drawn at the 30% probability level.

**Fig. 2.**
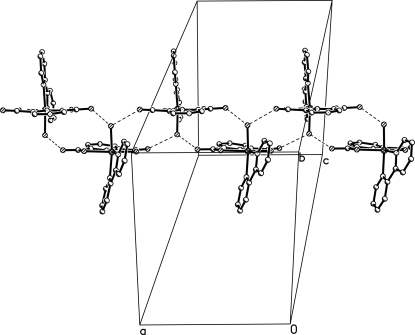
Part of the crystal structure of the title compound. Dashed lines indicate the donor-acceptor separation of O-H···O hydrogen bonds. The solvent water molecules are not shown.

## supplementary crystallographic information

### Comment

In general, bridging multifunctional organic ligands with N– and O-donors have been proven to be good candidates as versatile coordination sites and as hydrogen-bond acceptors and donors. Pyrazine-2,6-dicarboxylic acid (H_2_PZDC) has two carboxylic groups and two pyrazine nitrogen atoms, and can be used as hydrogen-bond acceptor as well as hydrogen-bond donor, which assists in the generation of supramolecular structures. Because of the structural character, we have used this ligand in our previous work to construct 3d, 4f and 3d-4f complexes (Wang *et al.*, 2006; Wang, Weng, *et al.*, 2007; Wang, Zheng & Jin, 2007; Wang, Zheng, *et al.*, 2007; Wang, Zheng *et al.*, 2008; Wang, Mu *et al.*, 2008). Generally, when coordinated to transition metal ions, PZDC adopts tridentate (ONO) mode and is involved in various hydrogen-bonding interactions. In a continuation of our work, we report synthesis and structure of the title complex.

The asymmetric unit of the title compound is shown in Fig. 1. In the crystal structure, intermolecular O-H···O (Fig. 2) and weak C-H···O hydrogen bonds link cations and water molecules into a three-dimensional network.

### Experimental

A mixture of H_2_PZDC (0.0204 g, 0.1 mmol), Ni(OAc)_2_.4H_2_O(0.0249 g, 0.1 mmol), 2,2'-bipyridine (0.0156 g, 0.1 mmol), deionized water (5 ml) and isopropyl alcohol (1 ml) was sealed in a Teflon-lined stainless steel vessel (23 ml) and heated at 413K for 4 days under autogenous pressure and then cooled slowly to room temperature. The solution was filtered and allowed to stand for four weeks at room temperature. Green crystals were obtained. Anal. calcd. for C_32_H_29_Ni_2_N_8_O_12.5_: C, 46.07; H, 3.38; N, 13.44. Found: C, 45.90; H, 3.49; N, 13.27%. IR (KBr pellet, cm^-1^): 3446br, 1663vs, 1630vs, 1475*m*, 1446 s, 1393*m*, 1339*m*, 1313w, 1192*m*, 1067*m*, 1024w, 791w, 766*m*, 749*m*.

### Refinement

All water H atoms were found in difference Fourier maps and were fixed during refinement at O–H distances of 0.85-0.86 Å, with *U*_iso_(H)=1.2 *U*_eq_(O). The H atoms of C–H and N–H groups were treated as riding, with C–H = 0.93 Å and N–H = 0.86 Å and *U*_iso_ (H) = 1.2 *U*_eq_(C,*N*).

### Crystal data

**Table:**

[Ni(C_6_H_2_N_2_O_4_)(C_10_H_8_N_2_)(H_2_O)]·1.25H_2_O	*F*(000) = 1732
*M**_r_* = 421.5	*D*_x_ = 1.626 Mg m^−^^3^
Monoclinic, *P*2_1_/*c*	Mo *K*α radiation, λ = 0.71073 Å
Hall symbol: -P 2ybc	Cell parameters from 5479 reflections
*a* = 10.7616 (12) Å	θ = 2.4–26.1°
*b* = 14.8677 (17) Å	µ = 1.17 mm^−^^1^
*c* = 21.933 (2) Å	*T* = 294 K
β = 101.015 (2)°	Block, green
*V* = 3444.6 (7) Å^3^	0.22 × 0.1 × 0.08 mm
*Z* = 8	

### Data collection

**Table:**

Bruker SMART CCD diffractometer	7030 independent reflections
Radiation source: fine-focus sealed tube	4740 reflections with *I* > 2σ(*I*)
graphite	*R*_int_ = 0.035
φ and ω scans	θ_max_ = 26.4°, θ_min_ = 1.7°
Absorption correction: multi-scan (SADABS; Sheldrick, 1996)	*h* = −11→13
*T*_min_ = 0.796, *T*_max_ = 0.871	*k* = −15→18
19122 measured reflections	*l* = −27→27

### Refinement

**Table:**

Refinement on *F*^2^	Primary atom site location: structure-invariant direct methods
Least-squares matrix: full	Secondary atom site location: difference Fourier map
*R*[*F*^2^ > 2σ(*F*^2^)] = 0.037	Hydrogen site location: inferred from neighbouring sites
*wR*(*F*^2^) = 0.102	H-atom parameters constrained
*S* = 1.04	*w* = 1/[σ^2^(*F*_o_^2^) + (0.042*P*)^2^ + 1.0664*P*] where *P* = (*F*_o_^2^ + 2*F*_c_^2^)/3
7030 reflections	(Δ/σ)_max_ < 0.001
496 parameters	Δρ_max_ = 0.31 e Å^−^^3^
0 restraints	Δρ_min_ = −0.26 e Å^−^^3^

### Special details

**Table:**

Geometry. All e.s.d.'s (except the e.s.d. in the dihedral angle between two l.s. planes) are estimated using the full covariance matrix. The cell e.s.d.'s are taken into account individually in the estimation of e.s.d.'s in distances, angles and torsion angles; correlations between e.s.d.'s in cell parameters are only used when they are defined by crystal symmetry. An approximate (isotropic) treatment of cell e.s.d.'s is used for estimating e.s.d.'s involving l.s. planes.
Refinement. Refinement of *F*^2^ against ALL reflections. The weighted *R*-factor *wR* and goodness of fit *S* are based on *F*^2^, conventional *R*-factors *R* are based on *F*, with *F* set to zero for negative *F*^2^. The threshold expression of *F*^2^ > σ(*F*^2^) is used only for calculating *R*-factors(gt) *etc*. and is not relevant to the choice of reflections for refinement. *R*-factors based on *F*^2^ are statistically about twice as large as those based on *F*, and *R*- factors based on ALL data will be even larger.

### Fractional atomic coordinates and isotropic or equivalent isotropic displacement parameters (Å^2^)

**Table:**

	*x*	*y*	*z*	*U*_iso_*/*U*_eq_	Occ. (<1)
Ni1	0.49882 (4)	0.13832 (3)	0.158117 (18)	0.04624 (12)	
Ni2	0.90485 (4)	0.54725 (3)	0.148920 (17)	0.04493 (12)	
O1	0.69996 (19)	0.12922 (15)	0.15470 (9)	0.0509 (5)	
O2	0.8901 (2)	0.17207 (17)	0.20621 (11)	0.0627 (6)	
O3	0.3425 (2)	0.17792 (16)	0.19965 (11)	0.0613 (6)	
O4	0.2998 (2)	0.24476 (17)	0.28546 (13)	0.0739 (7)	
O5	0.52042 (19)	0.01499 (15)	0.20153 (10)	0.0557 (6)	
H5A	0.4525	−0.0041	0.2114	0.067*	
H5B	0.5869	0.0128	0.2292	0.067*	
O6	1.10272 (19)	0.54558 (15)	0.15004 (9)	0.0504 (5)	
O7	1.2852 (2)	0.49209 (17)	0.20287 (10)	0.0633 (6)	
O8	0.73667 (19)	0.51540 (15)	0.18615 (10)	0.0556 (5)	
O9	0.6790 (2)	0.44246 (16)	0.26578 (12)	0.0682 (7)	
O10	0.91729 (19)	0.66902 (14)	0.19526 (9)	0.0529 (5)	
H10A	0.9788	0.6685	0.2263	0.063*	
H10B	0.8466	0.6873	0.2031	0.063*	
N1	0.5827 (2)	0.20244 (16)	0.23439 (11)	0.0444 (6)	
N2	0.6981 (3)	0.26952 (19)	0.34744 (12)	0.0604 (7)	
N3	0.4080 (3)	0.07653 (18)	0.07857 (12)	0.0550 (7)	
N4	0.4885 (2)	0.24284 (18)	0.09517 (12)	0.0519 (6)	
N5	0.9733 (2)	0.48052 (16)	0.22650 (11)	0.0442 (6)	
N6	1.0718 (3)	0.4020 (2)	0.33821 (12)	0.0604 (7)	
N7	0.8681 (2)	0.43822 (17)	0.08934 (11)	0.0461 (6)	
N8	0.8626 (2)	0.61198 (18)	0.06530 (11)	0.0489 (6)	
C1	0.7726 (3)	0.1651 (2)	0.19953 (14)	0.0473 (7)	
C2	0.7088 (3)	0.2040 (2)	0.24956 (13)	0.0438 (7)	
C3	0.7649 (3)	0.2362 (2)	0.30740 (14)	0.0534 (8)	
H3	0.8527	0.2345	0.3188	0.064*	
C4	0.5734 (4)	0.2687 (2)	0.32989 (16)	0.0594 (9)	
H4	0.5244	0.2930	0.3564	0.071*	
C5	0.5125 (3)	0.2329 (2)	0.27347 (14)	0.0496 (7)	
C6	0.3719 (3)	0.2181 (2)	0.25151 (18)	0.0577 (8)	
C7	0.3595 (4)	−0.0066 (3)	0.07472 (19)	0.0750 (11)	
H7	0.3617	−0.0396	0.1110	0.090*	
C8	0.3065 (4)	−0.0447 (3)	0.0188 (2)	0.0921 (14)	
H8	0.2738	−0.1027	0.0173	0.111*	
C9	0.3022 (4)	0.0034 (3)	−0.0349 (2)	0.0946 (15)	
H9	0.2684	−0.0219	−0.0733	0.114*	
C10	0.3488 (4)	0.0897 (3)	−0.03107 (17)	0.0792 (12)	
H10	0.3449	0.1240	−0.0668	0.095*	
C11	0.4016 (3)	0.1253 (2)	0.02637 (15)	0.0570 (8)	
C12	0.4508 (3)	0.2175 (2)	0.03524 (14)	0.0534 (8)	
C13	0.4574 (3)	0.2769 (3)	−0.01248 (17)	0.0683 (10)	
H13	0.4313	0.2588	−0.0535	0.082*	
C14	0.5023 (4)	0.3625 (3)	0.00065 (19)	0.0726 (11)	
H14	0.5083	0.4025	−0.0313	0.087*	
C15	0.5384 (4)	0.3881 (3)	0.06166 (19)	0.0695 (10)	
H15	0.5675	0.4461	0.0718	0.083*	
C16	0.5307 (3)	0.3264 (2)	0.10734 (17)	0.0586 (8)	
H16	0.5560	0.3437	0.1486	0.070*	
C17	1.1696 (3)	0.5046 (2)	0.19481 (14)	0.0476 (7)	
C18	1.0976 (3)	0.4675 (2)	0.24250 (13)	0.0453 (7)	
C19	1.1455 (3)	0.4261 (2)	0.29852 (15)	0.0552 (8)	
H19	1.2319	0.4146	0.3089	0.066*	
C20	0.9481 (3)	0.4179 (2)	0.32100 (15)	0.0548 (8)	
H20	0.8947	0.4032	0.3482	0.066*	
C21	0.8961 (3)	0.4554 (2)	0.26393 (14)	0.0461 (7)	
C22	0.7577 (3)	0.4730 (2)	0.23687 (16)	0.0504 (7)	
C23	0.8701 (3)	0.3514 (2)	0.10518 (15)	0.0541 (8)	
H23	0.8893	0.3365	0.1471	0.065*	
C24	0.8451 (3)	0.2828 (2)	0.06233 (16)	0.0604 (9)	
H24	0.8468	0.2230	0.0748	0.072*	
C25	0.8175 (3)	0.3058 (2)	0.00035 (15)	0.0626 (9)	
H25	0.8008	0.2611	−0.0298	0.075*	
C26	0.8147 (3)	0.3947 (2)	−0.01701 (15)	0.0574 (8)	
H26	0.7963	0.4106	−0.0588	0.069*	
C27	0.8394 (3)	0.4604 (2)	0.02840 (14)	0.0468 (7)	
C28	0.8390 (3)	0.5577 (2)	0.01516 (13)	0.0469 (7)	
C29	0.8194 (3)	0.5928 (3)	−0.04470 (15)	0.0605 (9)	
H29	0.7997	0.5548	−0.0788	0.073*	
C30	0.8294 (3)	0.6840 (3)	−0.05311 (18)	0.0685 (10)	
H30	0.8159	0.7084	−0.0929	0.082*	
C31	0.8596 (3)	0.7385 (3)	−0.00193 (18)	0.0658 (10)	
H31	0.8712	0.7999	−0.0066	0.079*	
C32	0.8723 (3)	0.7013 (2)	0.05624 (17)	0.0585 (8)	
H32	0.8882	0.7391	0.0907	0.070*	
O11	0.4752 (3)	0.56707 (19)	0.13832 (11)	0.0797 (8)	
H11A	0.4192	0.5445	0.1563	0.096*	
H11B	0.5514	0.5504	0.1514	0.096*	
O12	0.0915 (3)	0.1333 (3)	0.13827 (16)	0.1178 (12)	
H12A	0.1599	0.1044	0.1505	0.141*	
H12B	0.0622	0.1114	0.1026	0.141*	
O13	0.0028 (10)	0.0642 (8)	0.0279 (6)	0.235 (6)	0.50
H13A	0.0350	0.0114	0.0331	0.282*	0.50
H13B	−0.0776	0.0627	0.0204	0.282*	0.50

### Atomic displacement parameters (Å^2^)

**Table:**

	*U*^11^	*U*^22^	*U*^33^	*U*^12^	*U*^13^	*U*^23^
Ni1	0.0410 (2)	0.0513 (2)	0.0450 (2)	−0.00191 (17)	0.00470 (16)	−0.00111 (18)
Ni2	0.0391 (2)	0.0523 (2)	0.0422 (2)	0.00067 (17)	0.00479 (16)	−0.00043 (17)
O1	0.0459 (12)	0.0617 (14)	0.0452 (12)	−0.0003 (10)	0.0094 (10)	−0.0043 (10)
O2	0.0385 (13)	0.0865 (18)	0.0628 (14)	0.0001 (11)	0.0086 (10)	0.0134 (13)
O3	0.0426 (13)	0.0677 (15)	0.0741 (15)	−0.0030 (11)	0.0123 (11)	−0.0088 (13)
O4	0.0611 (16)	0.0648 (16)	0.107 (2)	−0.0064 (12)	0.0438 (15)	−0.0133 (14)
O5	0.0431 (12)	0.0632 (14)	0.0592 (13)	−0.0063 (10)	0.0061 (10)	0.0097 (11)
O6	0.0427 (12)	0.0635 (14)	0.0451 (11)	−0.0007 (10)	0.0092 (9)	0.0010 (10)
O7	0.0386 (13)	0.0897 (18)	0.0612 (14)	0.0015 (11)	0.0083 (10)	−0.0070 (13)
O8	0.0385 (12)	0.0664 (15)	0.0613 (14)	0.0016 (10)	0.0080 (10)	0.0019 (12)
O9	0.0531 (14)	0.0663 (16)	0.0934 (18)	0.0054 (11)	0.0350 (13)	0.0159 (13)
O10	0.0423 (12)	0.0601 (14)	0.0552 (12)	0.0041 (10)	0.0068 (10)	−0.0087 (10)
N1	0.0453 (15)	0.0449 (14)	0.0445 (13)	−0.0043 (11)	0.0122 (11)	0.0010 (11)
N2	0.074 (2)	0.0609 (18)	0.0449 (15)	−0.0132 (15)	0.0083 (14)	−0.0022 (13)
N3	0.0522 (16)	0.0561 (17)	0.0530 (16)	−0.0020 (13)	0.0004 (13)	−0.0071 (13)
N4	0.0491 (16)	0.0555 (17)	0.0501 (15)	0.0030 (12)	0.0071 (12)	0.0026 (12)
N5	0.0416 (14)	0.0484 (14)	0.0424 (13)	0.0000 (11)	0.0076 (11)	−0.0035 (11)
N6	0.065 (2)	0.0651 (19)	0.0488 (16)	0.0026 (14)	0.0050 (14)	0.0079 (14)
N7	0.0460 (14)	0.0533 (16)	0.0387 (13)	−0.0002 (11)	0.0072 (11)	0.0014 (11)
N8	0.0426 (14)	0.0539 (16)	0.0496 (15)	0.0022 (11)	0.0075 (12)	0.0024 (12)
C1	0.0461 (18)	0.0491 (18)	0.0470 (17)	0.0026 (14)	0.0095 (14)	0.0101 (14)
C2	0.0421 (17)	0.0447 (17)	0.0431 (16)	−0.0061 (13)	0.0046 (13)	0.0048 (13)
C3	0.057 (2)	0.0506 (19)	0.0494 (18)	−0.0080 (15)	0.0019 (15)	0.0025 (15)
C4	0.075 (3)	0.056 (2)	0.0527 (19)	−0.0087 (17)	0.0256 (18)	−0.0061 (16)
C5	0.054 (2)	0.0450 (17)	0.0525 (18)	−0.0047 (14)	0.0179 (15)	0.0016 (14)
C6	0.053 (2)	0.0485 (19)	0.076 (2)	−0.0003 (15)	0.0251 (18)	−0.0009 (17)
C7	0.076 (3)	0.065 (2)	0.076 (3)	−0.006 (2)	−0.006 (2)	−0.009 (2)
C8	0.100 (4)	0.066 (3)	0.096 (3)	−0.008 (2)	−0.017 (3)	−0.022 (3)
C9	0.104 (4)	0.096 (4)	0.072 (3)	0.008 (3)	−0.015 (2)	−0.036 (3)
C10	0.089 (3)	0.089 (3)	0.054 (2)	0.007 (2)	−0.001 (2)	−0.013 (2)
C11	0.0487 (19)	0.071 (2)	0.0497 (18)	0.0073 (16)	0.0052 (15)	−0.0071 (17)
C12	0.0469 (18)	0.067 (2)	0.0462 (17)	0.0096 (15)	0.0087 (14)	0.0018 (16)
C13	0.066 (2)	0.087 (3)	0.053 (2)	0.014 (2)	0.0145 (18)	0.0071 (19)
C14	0.071 (3)	0.075 (3)	0.076 (3)	0.011 (2)	0.024 (2)	0.025 (2)
C15	0.072 (3)	0.056 (2)	0.084 (3)	0.0040 (18)	0.021 (2)	0.011 (2)
C16	0.059 (2)	0.052 (2)	0.062 (2)	0.0026 (16)	0.0076 (17)	0.0027 (17)
C17	0.0385 (17)	0.0571 (19)	0.0473 (17)	−0.0020 (14)	0.0083 (13)	−0.0120 (15)
C18	0.0406 (17)	0.0494 (18)	0.0448 (16)	0.0029 (13)	0.0050 (13)	−0.0072 (13)
C19	0.0495 (19)	0.060 (2)	0.0525 (19)	0.0075 (15)	0.0003 (15)	−0.0005 (16)
C20	0.060 (2)	0.057 (2)	0.0487 (18)	−0.0043 (16)	0.0140 (16)	0.0001 (15)
C21	0.0472 (17)	0.0454 (17)	0.0465 (16)	0.0006 (14)	0.0109 (14)	−0.0052 (14)
C22	0.0450 (18)	0.0493 (19)	0.0585 (19)	0.0033 (14)	0.0144 (15)	−0.0025 (15)
C23	0.058 (2)	0.057 (2)	0.0465 (17)	−0.0006 (16)	0.0093 (15)	0.0054 (15)
C24	0.064 (2)	0.055 (2)	0.062 (2)	−0.0036 (16)	0.0100 (17)	−0.0016 (17)
C25	0.076 (2)	0.062 (2)	0.0496 (19)	−0.0089 (18)	0.0109 (17)	−0.0112 (16)
C26	0.059 (2)	0.070 (2)	0.0437 (17)	−0.0057 (17)	0.0100 (15)	−0.0025 (16)
C27	0.0369 (16)	0.0585 (19)	0.0451 (16)	−0.0010 (13)	0.0085 (13)	−0.0002 (14)
C28	0.0374 (16)	0.061 (2)	0.0433 (16)	0.0036 (14)	0.0095 (13)	0.0022 (14)
C29	0.067 (2)	0.071 (2)	0.0465 (18)	0.0098 (18)	0.0168 (16)	0.0062 (16)
C30	0.069 (2)	0.076 (3)	0.064 (2)	0.014 (2)	0.0234 (19)	0.021 (2)
C31	0.061 (2)	0.057 (2)	0.080 (3)	0.0065 (17)	0.0164 (19)	0.018 (2)
C32	0.052 (2)	0.054 (2)	0.067 (2)	0.0039 (15)	0.0062 (16)	0.0003 (17)
O11	0.0726 (18)	0.099 (2)	0.0685 (16)	0.0126 (15)	0.0163 (13)	0.0101 (15)
O12	0.087 (2)	0.157 (3)	0.106 (2)	0.005 (2)	0.0110 (19)	0.023 (2)
O13	0.156 (9)	0.262 (15)	0.298 (16)	−0.084 (9)	0.074 (10)	−0.068 (12)

### Geometric parameters (Å, °)

**Table:**

Ni1—N1	1.986 (2)	C7—C8	1.373 (5)
Ni1—N3	2.047 (3)	C7—H7	0.9300
Ni1—O5	2.059 (2)	C8—C9	1.371 (6)
Ni1—N4	2.067 (3)	C8—H8	0.9300
Ni1—O3	2.143 (2)	C9—C10	1.374 (6)
Ni1—O1	2.184 (2)	C9—H9	0.9300
Ni2—N5	1.986 (2)	C10—C11	1.384 (5)
Ni2—N8	2.044 (2)	C10—H10	0.9300
Ni2—O10	2.068 (2)	C11—C12	1.470 (5)
Ni2—N7	2.072 (2)	C12—C13	1.381 (5)
Ni2—O6	2.125 (2)	C13—C14	1.373 (5)
Ni2—O8	2.175 (2)	C13—H13	0.9300
O1—C1	1.253 (4)	C14—C15	1.374 (5)
O2—C1	1.249 (4)	C14—H14	0.9300
O3—C6	1.270 (4)	C15—C16	1.372 (5)
O4—C6	1.238 (4)	C15—H15	0.9300
O5—H5A	0.8500	C16—H16	0.9300
O5—H5B	0.8460	C17—C18	1.519 (4)
O6—C17	1.258 (4)	C18—C19	1.383 (4)
O7—C17	1.237 (3)	C19—H19	0.9300
O8—C22	1.261 (4)	C20—C21	1.387 (4)
O9—C22	1.237 (4)	C20—H20	0.9300
O10—H10A	0.8543	C21—C22	1.517 (4)
O10—H10B	0.8555	C23—C24	1.377 (4)
N1—C5	1.326 (4)	C23—H23	0.9300
N1—C2	1.334 (4)	C24—C25	1.378 (5)
N2—C4	1.324 (4)	C24—H24	0.9300
N2—C3	1.332 (4)	C25—C26	1.375 (5)
N3—C7	1.339 (4)	C25—H25	0.9300
N3—C11	1.346 (4)	C26—C27	1.384 (4)
N4—C16	1.332 (4)	C26—H26	0.9300
N4—C12	1.353 (4)	C27—C28	1.475 (4)
N5—C21	1.328 (4)	C28—C29	1.391 (4)
N5—C18	1.331 (4)	C29—C30	1.376 (5)
N6—C20	1.334 (4)	C29—H29	0.9300
N6—C19	1.334 (4)	C30—C31	1.372 (5)
N7—C23	1.337 (4)	C30—H30	0.9300
N7—C27	1.354 (4)	C31—C32	1.373 (5)
N8—C28	1.348 (4)	C31—H31	0.9300
N8—C32	1.350 (4)	C32—H32	0.9300
C1—C2	1.515 (4)	O11—H11A	0.8487
C2—C3	1.382 (4)	O11—H11B	0.8527
C3—H3	0.9300	O12—H12A	0.8501
C4—C5	1.390 (4)	O12—H12B	0.8505
C4—H4	0.9300	O13—H13A	0.8562
C5—C6	1.514 (5)	O13—H13B	0.8500
			
N1—Ni1—N3	177.78 (11)	C8—C7—H7	119.1
N1—Ni1—O5	92.57 (9)	C9—C8—C7	119.6 (4)
N3—Ni1—O5	89.30 (10)	C9—C8—H8	120.2
N1—Ni1—N4	99.17 (10)	C7—C8—H8	120.2
N3—Ni1—N4	79.18 (11)	C8—C9—C10	118.8 (4)
O5—Ni1—N4	164.84 (10)	C8—C9—H9	120.6
N1—Ni1—O3	77.15 (9)	C10—C9—H9	120.6
N3—Ni1—O3	101.53 (10)	C9—C10—C11	119.6 (4)
O5—Ni1—O3	94.39 (9)	C9—C10—H10	120.2
N4—Ni1—O3	97.53 (10)	C11—C10—H10	120.2
N1—Ni1—O1	76.38 (9)	N3—C11—C10	120.9 (4)
N3—Ni1—O1	104.94 (9)	N3—C11—C12	115.6 (3)
O5—Ni1—O1	86.31 (8)	C10—C11—C12	123.5 (3)
N4—Ni1—O1	87.17 (9)	N4—C12—C13	120.6 (3)
O3—Ni1—O1	153.53 (8)	N4—C12—C11	115.0 (3)
N5—Ni2—N8	170.60 (10)	C13—C12—C11	124.4 (3)
N5—Ni2—O10	92.16 (9)	C14—C13—C12	120.0 (4)
N8—Ni2—O10	90.64 (9)	C14—C13—H13	120.0
N5—Ni2—N7	98.30 (10)	C12—C13—H13	120.0
N8—Ni2—N7	79.60 (10)	C13—C14—C15	119.0 (4)
O10—Ni2—N7	168.92 (9)	C13—C14—H14	120.5
N5—Ni2—O6	77.14 (9)	C15—C14—H14	120.5
N8—Ni2—O6	93.80 (9)	C16—C15—C14	118.7 (4)
O10—Ni2—O6	91.94 (8)	C16—C15—H15	120.6
N7—Ni2—O6	93.92 (9)	C14—C15—H15	120.6
N5—Ni2—O8	76.62 (9)	N4—C16—C15	122.9 (3)
N8—Ni2—O8	112.40 (9)	N4—C16—H16	118.6
O10—Ni2—O8	89.23 (8)	C15—C16—H16	118.6
N7—Ni2—O8	89.66 (9)	O7—C17—O6	126.4 (3)
O6—Ni2—O8	153.76 (8)	O7—C17—C18	118.6 (3)
C1—O1—Ni1	114.87 (19)	O6—C17—C18	115.0 (3)
C6—O3—Ni1	115.4 (2)	N5—C18—C19	119.0 (3)
Ni1—O5—H5A	112.9	N5—C18—C17	112.6 (3)
Ni1—O5—H5B	111.8	C19—C18—C17	128.4 (3)
H5A—O5—H5B	117.0	N6—C19—C18	122.2 (3)
C17—O6—Ni2	115.92 (19)	N6—C19—H19	118.9
C22—O8—Ni2	114.71 (19)	C18—C19—H19	118.9
Ni2—O10—H10A	110.5	N6—C20—C21	122.3 (3)
Ni2—O10—H10B	113.6	N6—C20—H20	118.8
H10A—O10—H10B	115.2	C21—C20—H20	118.8
C5—N1—C2	120.7 (3)	N5—C21—C20	118.6 (3)
C5—N1—Ni1	119.0 (2)	N5—C21—C22	113.1 (3)
C2—N1—Ni1	119.7 (2)	C20—C21—C22	128.2 (3)
C4—N2—C3	116.7 (3)	O9—C22—O8	127.6 (3)
C7—N3—C11	119.2 (3)	O9—C22—C21	116.9 (3)
C7—N3—Ni1	125.8 (3)	O8—C22—C21	115.4 (3)
C11—N3—Ni1	115.0 (2)	N7—C23—C24	123.1 (3)
C16—N4—C12	118.8 (3)	N7—C23—H23	118.4
C16—N4—Ni1	126.5 (2)	C24—C23—H23	118.4
C12—N4—Ni1	114.0 (2)	C23—C24—C25	117.8 (3)
C21—N5—C18	120.8 (3)	C23—C24—H24	121.1
C21—N5—Ni2	119.8 (2)	C25—C24—H24	121.1
C18—N5—Ni2	119.2 (2)	C26—C25—C24	120.1 (3)
C20—N6—C19	117.0 (3)	C26—C25—H25	120.0
C23—N7—C27	118.8 (3)	C24—C25—H25	120.0
C23—N7—Ni2	126.9 (2)	C25—C26—C27	119.2 (3)
C27—N7—Ni2	114.3 (2)	C25—C26—H26	120.4
C28—N8—C32	118.5 (3)	C27—C26—H26	120.4
C28—N8—Ni2	115.2 (2)	N7—C27—C26	121.0 (3)
C32—N8—Ni2	125.8 (2)	N7—C27—C28	115.1 (3)
O2—C1—O1	126.4 (3)	C26—C27—C28	123.9 (3)
O2—C1—C2	118.2 (3)	N8—C28—C29	121.1 (3)
O1—C1—C2	115.4 (3)	N8—C28—C27	115.7 (3)
N1—C2—C3	118.7 (3)	C29—C28—C27	123.2 (3)
N1—C2—C1	113.1 (2)	C30—C29—C28	119.6 (3)
C3—C2—C1	128.2 (3)	C30—C29—H29	120.2
N2—C3—C2	122.6 (3)	C28—C29—H29	120.2
N2—C3—H3	118.7	C31—C30—C29	119.0 (3)
C2—C3—H3	118.7	C31—C30—H30	120.5
N2—C4—C5	122.8 (3)	C29—C30—H30	120.5
N2—C4—H4	118.6	C30—C31—C32	119.2 (3)
C5—C4—H4	118.6	C30—C31—H31	120.4
N1—C5—C4	118.4 (3)	C32—C31—H31	120.4
N1—C5—C6	113.8 (3)	N8—C32—C31	122.4 (3)
C4—C5—C6	127.6 (3)	N8—C32—H32	118.8
O4—C6—O3	127.7 (3)	C31—C32—H32	118.8
O4—C6—C5	117.7 (3)	H11A—O11—H11B	116.8
O3—C6—C5	114.6 (3)	H12A—O12—H12B	104.0
N3—C7—C8	121.8 (4)	H13A—O13—H13B	111.9
N3—C7—H7	119.1		
			
N1—Ni1—O1—C1	0.7 (2)	C2—N1—C5—C6	−174.5 (3)
N3—Ni1—O1—C1	178.8 (2)	Ni1—N1—C5—C6	−3.3 (3)
O5—Ni1—O1—C1	−92.9 (2)	N2—C4—C5—N1	−3.4 (5)
N4—Ni1—O1—C1	100.8 (2)	N2—C4—C5—C6	172.2 (3)
O3—Ni1—O1—C1	−0.4 (3)	Ni1—O3—C6—O4	−177.4 (3)
N1—Ni1—O3—C6	−2.6 (2)	Ni1—O3—C6—C5	1.7 (4)
N3—Ni1—O3—C6	179.2 (2)	N1—C5—C6—O4	−180.0 (3)
O5—Ni1—O3—C6	89.0 (2)	C4—C5—C6—O4	4.2 (5)
N4—Ni1—O3—C6	−100.3 (2)	N1—C5—C6—O3	0.9 (4)
O1—Ni1—O3—C6	−1.5 (4)	C4—C5—C6—O3	−174.9 (3)
N5—Ni2—O6—C17	1.5 (2)	C11—N3—C7—C8	1.9 (6)
N8—Ni2—O6—C17	−175.9 (2)	Ni1—N3—C7—C8	−176.1 (3)
O10—Ni2—O6—C17	93.3 (2)	N3—C7—C8—C9	−0.3 (7)
N7—Ni2—O6—C17	−96.1 (2)	C7—C8—C9—C10	−1.4 (7)
O8—Ni2—O6—C17	1.1 (3)	C8—C9—C10—C11	1.6 (7)
N5—Ni2—O8—C22	0.8 (2)	C7—N3—C11—C10	−1.8 (5)
N8—Ni2—O8—C22	178.0 (2)	Ni1—N3—C11—C10	176.5 (3)
O10—Ni2—O8—C22	−91.6 (2)	C7—N3—C11—C12	176.8 (3)
N7—Ni2—O8—C22	99.4 (2)	Ni1—N3—C11—C12	−5.0 (4)
O6—Ni2—O8—C22	1.2 (3)	C9—C10—C11—N3	0.0 (6)
O5—Ni1—N1—C5	−90.7 (2)	C9—C10—C11—C12	−178.4 (4)
N4—Ni1—N1—C5	98.9 (2)	C16—N4—C12—C13	0.8 (5)
O3—Ni1—N1—C5	3.2 (2)	Ni1—N4—C12—C13	−170.4 (2)
O1—Ni1—N1—C5	−176.3 (2)	C16—N4—C12—C11	−177.9 (3)
O5—Ni1—N1—C2	80.6 (2)	Ni1—N4—C12—C11	10.9 (3)
N4—Ni1—N1—C2	−89.8 (2)	N3—C11—C12—N4	−4.1 (4)
O3—Ni1—N1—C2	174.5 (2)	C10—C11—C12—N4	174.4 (3)
O1—Ni1—N1—C2	−5.0 (2)	N3—C11—C12—C13	177.3 (3)
O5—Ni1—N3—C7	16.3 (3)	C10—C11—C12—C13	−4.2 (5)
N4—Ni1—N3—C7	−173.6 (3)	N4—C12—C13—C14	0.0 (5)
O3—Ni1—N3—C7	−78.0 (3)	C11—C12—C13—C14	178.5 (3)
O1—Ni1—N3—C7	102.3 (3)	C12—C13—C14—C15	−1.1 (6)
O5—Ni1—N3—C11	−161.8 (2)	C13—C14—C15—C16	1.4 (6)
N4—Ni1—N3—C11	8.3 (2)	C12—N4—C16—C15	−0.4 (5)
O3—Ni1—N3—C11	103.9 (2)	Ni1—N4—C16—C15	169.5 (3)
O1—Ni1—N3—C11	−75.8 (2)	C14—C15—C16—N4	−0.7 (6)
N1—Ni1—N4—C16	0.7 (3)	Ni2—O6—C17—O7	176.5 (3)
N3—Ni1—N4—C16	179.2 (3)	Ni2—O6—C17—C18	−3.2 (3)
O5—Ni1—N4—C16	−139.6 (3)	C21—N5—C18—C19	0.9 (4)
O3—Ni1—N4—C16	78.8 (3)	Ni2—N5—C18—C19	175.9 (2)
O1—Ni1—N4—C16	−75.0 (3)	C21—N5—C18—C17	−177.4 (3)
N1—Ni1—N4—C12	171.0 (2)	Ni2—N5—C18—C17	−2.5 (3)
N3—Ni1—N4—C12	−10.5 (2)	O7—C17—C18—N5	−176.0 (3)
O5—Ni1—N4—C12	30.7 (5)	O6—C17—C18—N5	3.7 (4)
O3—Ni1—N4—C12	−110.8 (2)	O7—C17—C18—C19	5.8 (5)
O1—Ni1—N4—C12	95.3 (2)	O6—C17—C18—C19	−174.5 (3)
O10—Ni2—N5—C21	84.3 (2)	C20—N6—C19—C18	1.4 (5)
N7—Ni2—N5—C21	−92.1 (2)	N5—C18—C19—N6	−2.8 (5)
O6—Ni2—N5—C21	175.8 (2)	C17—C18—C19—N6	175.3 (3)
O8—Ni2—N5—C21	−4.4 (2)	C19—N6—C20—C21	1.7 (5)
O10—Ni2—N5—C18	−90.8 (2)	C18—N5—C21—C20	2.1 (4)
N7—Ni2—N5—C18	92.9 (2)	Ni2—N5—C21—C20	−172.9 (2)
O6—Ni2—N5—C18	0.7 (2)	C18—N5—C21—C22	−178.2 (3)
O8—Ni2—N5—C18	−179.5 (2)	Ni2—N5—C21—C22	6.9 (3)
N5—Ni2—N7—C23	10.9 (3)	N6—C20—C21—N5	−3.5 (5)
N8—Ni2—N7—C23	−178.4 (3)	N6—C20—C21—C22	176.8 (3)
O10—Ni2—N7—C23	−149.8 (4)	Ni2—O8—C22—O9	−175.5 (3)
O6—Ni2—N7—C23	88.4 (3)	Ni2—O8—C22—C21	2.4 (3)
O8—Ni2—N7—C23	−65.5 (3)	N5—C21—C22—O9	172.2 (3)
N5—Ni2—N7—C27	−169.2 (2)	C20—C21—C22—O9	−8.1 (5)
N8—Ni2—N7—C27	1.6 (2)	N5—C21—C22—O8	−5.9 (4)
O10—Ni2—N7—C27	30.2 (6)	C20—C21—C22—O8	173.9 (3)
O6—Ni2—N7—C27	−91.6 (2)	C27—N7—C23—C24	0.5 (5)
O8—Ni2—N7—C27	114.4 (2)	Ni2—N7—C23—C24	−179.6 (2)
O10—Ni2—N8—C28	−177.6 (2)	N7—C23—C24—C25	0.3 (5)
N7—Ni2—N8—C28	−2.9 (2)	C23—C24—C25—C26	−0.5 (5)
O6—Ni2—N8—C28	90.4 (2)	C24—C25—C26—C27	−0.2 (5)
O8—Ni2—N8—C28	−88.2 (2)	C23—N7—C27—C26	−1.1 (4)
O10—Ni2—N8—C32	11.3 (3)	Ni2—N7—C27—C26	178.9 (2)
N7—Ni2—N8—C32	−174.0 (3)	C23—N7—C27—C28	179.8 (3)
O6—Ni2—N8—C32	−80.7 (3)	Ni2—N7—C27—C28	−0.1 (3)
O8—Ni2—N8—C32	100.7 (3)	C25—C26—C27—N7	1.0 (5)
Ni1—O1—C1—O2	−176.4 (2)	C25—C26—C27—C28	179.9 (3)
Ni1—O1—C1—C2	3.1 (3)	C32—N8—C28—C29	−2.8 (4)
C5—N1—C2—C3	1.3 (4)	Ni2—N8—C28—C29	−174.6 (2)
Ni1—N1—C2—C3	−169.8 (2)	C32—N8—C28—C27	175.5 (3)
C5—N1—C2—C1	179.1 (3)	Ni2—N8—C28—C27	3.6 (3)
Ni1—N1—C2—C1	7.9 (3)	N7—C27—C28—N8	−2.3 (4)
O2—C1—C2—N1	172.5 (3)	C26—C27—C28—N8	178.7 (3)
O1—C1—C2—N1	−7.0 (4)	N7—C27—C28—C29	175.9 (3)
O2—C1—C2—C3	−10.0 (5)	C26—C27—C28—C29	−3.1 (5)
O1—C1—C2—C3	170.5 (3)	N8—C28—C29—C30	2.7 (5)
C4—N2—C3—C2	1.5 (5)	C27—C28—C29—C30	−175.4 (3)
N1—C2—C3—N2	−3.1 (5)	C28—C29—C30—C31	0.5 (5)
C1—C2—C3—N2	179.5 (3)	C29—C30—C31—C32	−3.4 (5)
C3—N2—C4—C5	1.7 (5)	C28—N8—C32—C31	−0.3 (5)
C2—N1—C5—C4	1.7 (4)	Ni2—N8—C32—C31	170.5 (3)
Ni1—N1—C5—C4	172.9 (2)	C30—C31—C32—N8	3.5 (5)

### Hydrogen-bond geometry (Å, °)

**Table:**

*D*—H···*A*	*D*—H	H···*A*	*D*···*A*	*D*—H···*A*
C32—H32···O10	0.93	2.48	3.033 (4)	118
C31—H31···O13^i^	0.93	2.53	3.384 (11)	153
C29—H29···O6^ii^	0.93	2.53	3.318 (4)	143
C26—H26···O6^ii^	0.93	2.54	3.329 (4)	143
C14—H14···O11^i^	0.93	2.43	3.275 (5)	151
C9—H9···O1^iii^	0.93	2.47	3.281 (5)	146
C7—H7···O5	0.93	2.50	3.000 (4)	114
O13—H13A···O13^iv^	0.86	1.73	2.26 (2)	118
O12—H12B···O13	0.85	1.79	2.636 (12)	179
O12—H12A···O3	0.85	2.33	2.856 (4)	121
O11—H11B···O8	0.85	2.06	2.912 (3)	177
O11—H11A···O7^v^	0.85	2.07	2.921 (4)	178
O10—H10B···O4^vi^	0.86	1.85	2.699 (3)	170
O10—H10A···O2^vii^	0.85	1.84	2.694 (3)	178
O5—H5B···O7^viii^	0.85	1.85	2.686 (3)	169
O5—H5A···O9^ix^	0.85	1.78	2.621 (3)	173

Symmetry codes: (i) −*x*+1, −*y*+1, −*z*; (ii) −*x*+2, −*y*+1, −*z*; (iii) −*x*+1, −*y*, −*z*; (iv) −*x*, −*y*, −*z*; (v) *x*−1, *y*, *z*; (vi) −*x*+1, *y*+1/2, −*z*+1/2; (vii) −*x*+2, *y*+1/2, −*z*+1/2; (viii) −*x*+2, *y*−1/2, −*z*+1/2; (ix) −*x*+1, *y*−1/2, −*z*+1/2.
